# Induced dual-target rebalance simultaneously enhances efficient therapeutical efficacy in tumors

**DOI:** 10.1038/s41420-024-02018-y

**Published:** 2024-05-23

**Authors:** Xiaoyu Zhang, Tianyi Ding, Fan Yang, Haowen Xu, Jixing Zhang, Yiran Bai, Yibing Shi, Jiaqi Yang, Chaoqun Chen, He Zhang

**Affiliations:** 1 State Key Laboratory of Cardiology and Medical Innovation Center, Institute for Regenerative Medicine, Shanghai East Hospital, Frontier Science Research Center for Stem Cells, School of Life Science and Technology, Tongji University, Shanghai, 200092 China; 2 Jiangxi Province Key Laboratory of Organ Development and Epigenetics, Clinical Medical Research Center, Affiliated Hospital of Jinggangshan University, Medical Department of Jinggangshan University, Ji’an, 343009 China; 3 School of Life Science, Jinggangshan University, Ji’an, 343009 China

**Keywords:** Targeted therapies, Cancer epigenetics

## Abstract

Multiple gene abnormalities are major drivers of tumorigenesis. NF-κB p65 overactivation and cGAS silencing are important triggers and genetic defects that accelerate tumorigenesis. However, the simultaneous correction of NF-κB p65 and cGAS abnormalities remains to be further explored. Here, we propose a novel **I**nduced **D**ual-**T**arget **R**ebalance (IDTR) strategy for simultaneously correcting defects in cGAS and NF-κB p65. By using our IDTR approach, we showed for the first time that oncolytic adenovirus H101 could reactivate silenced cGAS, while silencing *GAU1* long noncoding RNA (lncRNA) inhibited NF-κB p65 overactivation, resulting in efficient in vitro and in vivo antitumor efficacy in colorectal tumors. Intriguingly, we further demonstrated that oncolytic adenoviruses reactivated cGAS by promoting H3K4 trimethylation of the *cGAS* promoter. In addition, silencing *GAU1* using antisense oligonucleotides significantly reduced H3K27 acetylation at the *NF-κB p65* promoter and inhibited *NF-κB p65* transcription. Our study revealed an aberrant therapeutic mechanism underlying two tumor defects, cGAS and NF-κB p65, and provided an alternative IDTR approach based on oncolytic adenovirus and antisense oligonucleotides for efficient therapeutic efficacy in tumors.

## Introduction

As a multigene disease, malignant tumors are usually accompanied by the activation of multiple oncogenes and the inactivation of various tumor suppressors [1]. Conventional single-target therapies primarily target one signaling pathway, however, tumor cells frequently acquire resistance by activating alternative pathways [2]. In lung adenocarcinoma, *KRAS* mutations accompanied by *Stk11/Lkb1* deletion were found to promote resistance to PD-1/PD-L1 inhibitors [3]. Consequently, multitargeted therapies are emerging as a novel trend in the field of cancer therapeutics aimed at simultaneously targeting multiple signaling pathways [4, 5]. For instance, in early-stage melanoma, the pathologic response rate for PD1 therapy was only 30–33%, whereas the pathologic response rate for CTLA4 + PD1 combined therapy was 71–80% [6, 7]. In addition, drugs that simultaneously target EGFR and HER2 have been used in pancreatic ductal adenocarcinoma and have shown highly efficient antitumor effects [8]. Therefore, concurrently targeting multiple tumor defects is an effective and attractive strategy for tumor therapy.

Cyclic GMP-AMP synthase (cGAS) is located in the cytoplasm and recognizes abnormal DNA in cellular compartments, catalyzing the synthesis of cyclic GMP-AMP (cGAMP) by GTP and ATP [9]. The activation of cGAS plays a major role in preventing early neoplastic progression by upregulating the expression of various inflammatory genes, including Type 1 IFNs [10]. However, cGAS silencing suppresses the innate immune response and enhances tumor susceptibility [11]. In colorectal tumors, cGAS deficiency impairs the intestinal epithelial barrier and exacerbates inflammation, leading to a more severe stage of tumor malignancy [12, 13]. On the other hand, NF-κB is another critical transcription factor involved in controlling DNA transcription, cytokine production and cell survival [14]. NF-κB p65 is the core subunit of the NF-κB complex. Overactivation of NF-κB p65 promotes tumorigenesis and is common in most tumors [15]. In non-small cell lung cancer (NSCLC), overactivation of NF-κB p65 binds to the *PD-L1* promoter and activates *PD-L1* transcription, leading to immune escape [16]. It is common for cGAS inactivation and NF-κB p65 overactivation to occur simultaneously in tumors. Therefore, simultaneous reactivation of silenced cGAS and inhibition of NF-κB p65 may be a potential and interesting strategy for antitumor therapy.

Recently, human DNA and RNA viruses have been shown to be involved in the activation of host genes [17]. For instance, Rta (KSHV) induces IL10 expression through interactions with the host transcription factors SP1 and SP3 [18]. The viral bZIP transcription factor Zta directly reprograms cellular gene expression through distal elements and promotes transcription of *TGFB1* genes [19]. These studies suggest that reactivation of silenced cGAS via a virus-oriented method could be an interesting approach. On the other hand, studies have reported that *NKILA* lncRNA could be involved in inhibiting NF-κB protein activity by interfering with the NF-κB/Snail signaling pathway in NSCLC cells [20]. Thus, inhibiting the overactivation of NF-κB p65 by using a lncRNA-oriented method is likely to be an alternative avenue for treating tumors. Therefore, we further propose the use of induced dual-target rebalance (IDTR) to simultaneously correct cGAS and NF-κB p65 abnormalities by using viral and lncRNA-oriented combinations to improve overall antitumor efficacy.

In this study, we successfully established a novel, efficient antitumor approach for IDTR based on the combination of oncolytic adenovirus and *GAU1* lncRNA. We further demonstrated an alternative therapeutic mechanism underlying cGAS and NF-κB p65 abnormalities and identified a potential and interesting antitumor therapeutic strategy.

## Results

### Identifying tumor models for simultaneously correcting cGAS and NF-κB p65 abnormalities

**Fig. 1:**
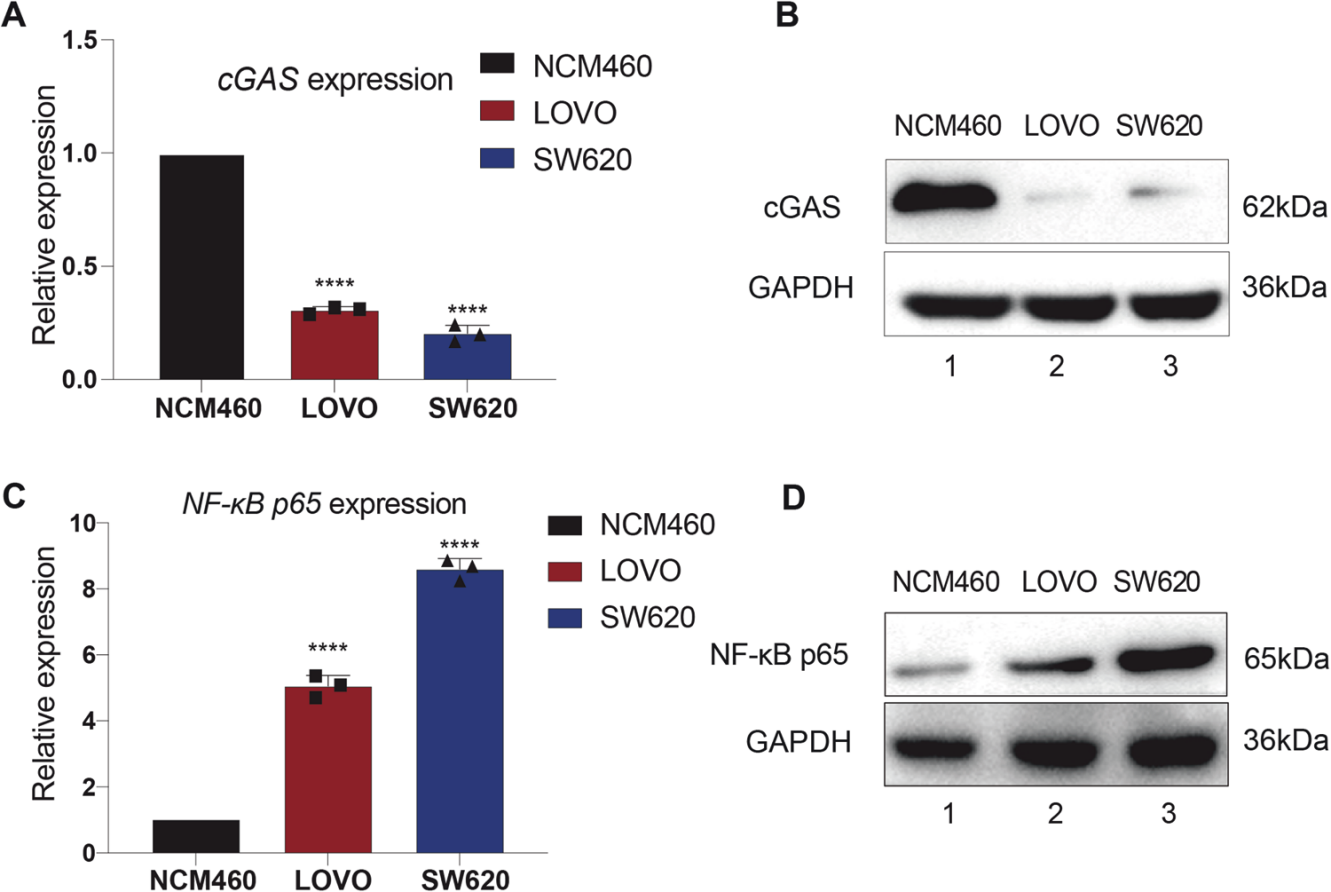
Identifying tumor models for simultaneously correcting cGAS and NF-κB p65 abnormalities. **A** Real-time PCR revealed the *cGAS* mRNA expression in tumor cells (SW620 and LOVO). *GAPDH* served as the internal control for normalization. Data is presented as mean ± SD, and differences between two groups were calculated using an unpaired two-tailed *t-*test. N = 3, ****P < 0.0001 compared to normal enterocyte NCM460. **B** Western blot analysis demonstrated the cGAS protein expression in tumor cells SW620 and LOVO (Lanes 2–3) in comparison to normal cell line NCM460 (Lane 1). **C** Real-time PCR showed the *NF-κB p65* mRNA expression in tumor cells (SW620 and LOVO tumor cells). *GAPDH* was used as the internal control for normalization. Data is presented as mean ± SD, and differences between two groups were calculated using an unpaired two-tailed *t*-test. N = 3, ****P < 0.0001 compared to normal enterocytes NCM460. **D** Western blot showed the expression of NF-κB p65 protein in SW620 and LOVO tumor cells (Lanes 2–3), compared to normal cells NCM460 (Lane 1).

To validate our hypothesis, we initially established a tumor model with both cGAS and NF-κB p65 abnormalities. We selected two representative human colorectal cancer cell lines, SW620 and LOVO, to detect the expression of cGAS and NF-κB p65. As expected, we observed that the expression of *cGAS* was significantly lower in SW620 and LOVO tumor cells than in normal enterocytes NCM460 (Fig. 1A). Western blot analysis also revealed that cGAS was expressed at low levels in two colorectal cancer cell lines (Fig. 1B, Lanes 2–3 and Original full length western blots image 1). Furthermore, we examined NF-κB p65 expression. As expected, real-time PCR analysis indicated a remarkable increase in the RNA level of *NF-κB p65* in SW620 and LOVO tumor cells compared to NCM460 control cells (Fig. 1C). These findings were further confirmed by western blot analysis, which revealed elevated levels of NF-κB p65 protein in SW620 and LOVO tumor cells (Fig. 1D, Lanes 2–3 and Original full length western blots image 1). Taken together, these data indicate that cGAS silencing and NF-κB p65 overactivation occurred simultaneously in SW620 and LOVO tumor cells. We next used these two types of tumor cells as our tumor models for testing the IDTR strategy.

### The oncolytic adenovirus H101 significantly activated cGAS expression

**Fig. 2:**
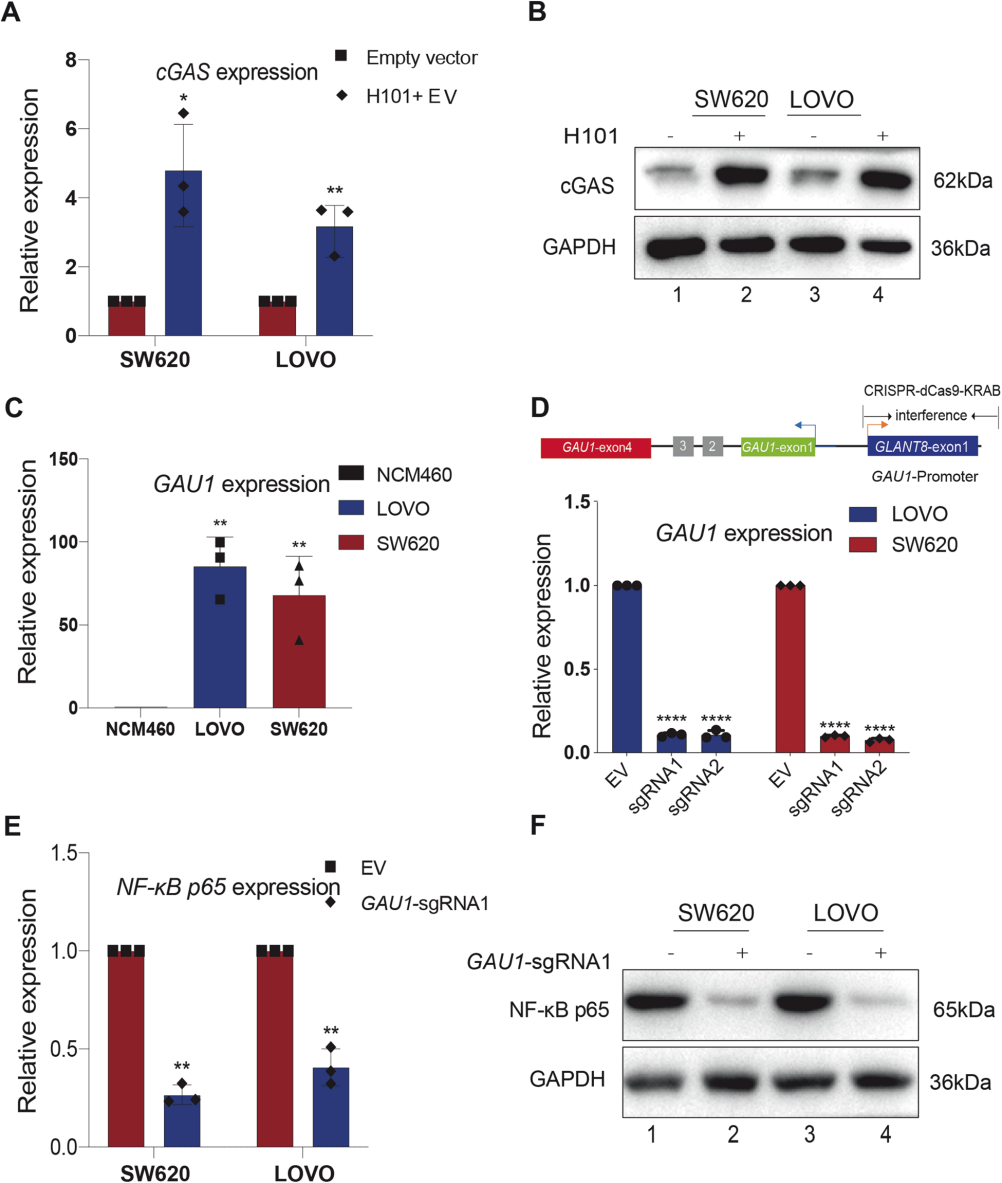
H101 activates cGAS expression and *GAU1* lncRNA inhibits over-expression of NF-κB p65*.* **A** Real-time PCR analysis revealed the expression of *cGAS* mRNA levels following H101 infection in SW620 and LOVO cells. All experiments were conducted 72 hours after H101 infection (MOI = 100). *GAPDH* was utilized as the internal control for normalization. The data are presented as the mean ± SD, and the differences between the two groups were calculated using an unpaired two-tailed *t*-test. Statistical significance was denoted as **P < 0.01 and *P < 0.05, N = 3. **B** Western blot analysis showed the level of cGAS protein after 72 hours of H101 infection in SW620 and LOVO cells. **C** Real-time PCR showed that the *GAU1* lncRNA expression in tumor cells. Data are presented as the mean ± SD and the differences between two groups were calculated by unpaired two-tailed *t-*test. N = 3, **P < 0.01 compared with NCM460. **D** Real-time PCR analysis showed the expression of *GAU1* knockdown by CRISPR/dCas9-KRBA method in SW620 and LOVO cells. Data are presented as the mean ± SD and the differences between two groups were calculated by unpaired two-tailed *t-*test. N = 3, ****P < 0.0001 compared with empty vector (Empty Vector, EV: cells transfected with the empty dCas9-KRAB vector). **E** Real-time PCR showed the *NF-κB p65* mRNA levels in SW620 and LOVO tumor cells after *GAU1* knockdown. *GAPDH* was used as the internal control for normalization. Data are presented as the mean ± SD and the differences between two groups were calculated by unpaired two-tailed *t*-test. N = 3, **P < 0.01. **F** Western blot confirmed the expression of NF-κB p65 protein after *GAU1* knockdown in SW620 and LOVO tumor cells.

Next, we attempted to reactivate silenced cGAS by using the oncolytic adenovirus H101 in SW620 and LOVO tumor cells. After infecting tumor cells with a multiplicity of infection (MOI) of 100 for 72 hours, we conducted real-time PCR experiments to investigate the gene expression of *cGAS*. As expected, we observed significant upregulation of the RNA level of *cGAS* compared to that in the normal group. Specifically, *cGAS* expression in SW620 and LOVO cells increased 3.9-fold and 3.5-fold, respectively, after H101 treatment (Fig. 2A). We also utilized western blot techniques to assess alterations in the expression of the cGAS protein. After 72 hours of exposure to H101, the tumor cells exhibited a substantial increase in the cGAS protein level compared to that in the control group (Fig. 2B and Original full length western blots image 2). These findings indicate that oncolytic adenovirus H101 can effectively reactivate silenced cGAS in SW620 and LOVO tumor cells.

### *GAU1* can inhibit the overexpression of NF-κB p65

We further explored a lncRNA-oriented method to specifically inhibit the overexpression of NF-κB p65 in tumors. *GAU1* is a long noncoding RNA that we discovered and named during our early research, and we aimed to further explore its potential in regulating NF-κB p65. We found that the *GAU1* lncRNA was highly expressed in SW620 and LOVO tumor cells (Fig. 2C). To elucidate the regulatory role of *GAU1* lncRNA in *NF-κB p65* transcriptional regulation, we utilized CRISPR/dCas9-KRAB technology to design a gene knockdown system without deleting genomic DNA. We employed two specific sgRNAs to target SW620 and LOVO cells and successfully reduced *GAU1* expression to a range of 6.3–13.7% of the original *GAU1* level. Specifically, *GAU1* expression in LOVO cells decreased to 9.2–13.7% of its original level, while *GAU1* expression decreased to 6.3–10.5% of its original level in SW620 cells (Fig. 2D). Subsequently, we conducted real-time PCR analysis to assess the expression of *NF-κB p65* following *GAU1* knockdown. Intriguingly, we observed substantial 66.4% and 66.6% reductions in *NF-κB p65* expression after *GAU1* knockdown in LOVO and SW620 cells, respectively (Fig. 2E). Furthermore, western blot analysis revealed that the protein levels of NF-κB p65 were notably reduced after *GAU1* knockdown (Fig. 2F and Original full length western blots image 2). These data suggest that *GAU1* is a key factor in promoting NF-κB p65 expression and that *GAU1* silencing could effectively inhibit the overexpression of NF-κB p65.

### The IDTR strategy inhibits tumor growth in colorectal tumor cells

**Fig. 3:**
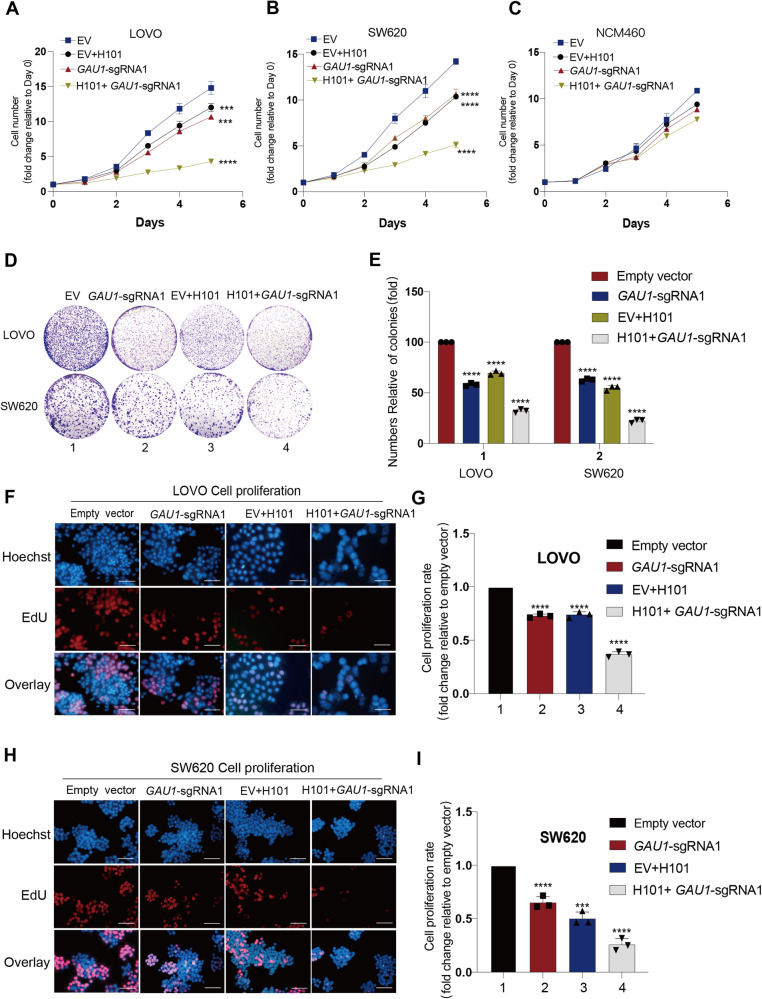
The IDTR strategy inhibits tumor growth in colorectal tumor cells. **A** Cell growth rate was measured by CCK8 assay on days 0,1, 2, 3, 4 and 5 after the cells were treated with EV (Square), H101(Cycle), *GAU1*-sgRNA (Triangle) and H101 + *GAU1*-sgRNA (Inverted triangle) in the LOVO cells. **B** Cell growth rate was measured by CCK8 assay in the SW620 cells. Experimental methods and annotations were described above. **C** Cell growth rate was measured by CCK8 assay in the NCM460 normal cells. Experimental methods and annotations were described above. **D** Cloning formation assay was conducted to assess the colony formation ability of EV, EV + H101, *GAU1*-sgRNA, H101 + *GAU1*-sgRNA. **E** Quantification of visible colonies was performed. The colony count in the empty vector group was set as 100%. All experiments were performed in triplicate, and the relative colony formation rates were showed as the mean ± SEM. N = 3, ****P < 0.0001. **F** Representative micrographs from LOVO cells treated with 10 *μ*M EdU for 4 hours were provided. Each type of cell was categorized based on treatment: EV (Lane 1), H101(Lane 2), *GAU1*-sgRNA (Lane 3) and H101 + *GAU1*-sgRNA (Lane 4) groups. Blue: Hoechst 33342 staining of nuclear; RED: Alexa Fluor 555 staining of proliferating cells. The scale bar was 5 mm. **G** LOVO cells proliferation quantification. The proportion of proliferating cells in the empty vector group was set to 1. The proportion of proliferating cells in the other experimental groups was compared to that in the empty vector group. Data were presented as the mean ± SD and the differences between two groups were calculated by unpaired two-tailed *t*-test. N = 3, ****P < 0.0001. **H** Representative micrographs from SW620 cells treated with 10 *μ*M EdU for 4 hours were provided. Experimental methods and groupings were described above. The scale bar was 5 mm. **I** SW620 cells proliferation quantification. Experimental methods, grouping and data processing were described above. Data were presented as the mean ± SD and the differences between two groups were calculated by unpaired two-tailed *t*-test. N = 3, ****P < 0.0001 and ***P < 0.001.

Given that oncolytic adenovirus H101 reactivated cGAS and *GAU1* knockdown inhibited NF-κB p65 overexpression, we next tested the antitumor efficacy of IDTR by combining oncolytic adenovirus H101 infection and *GAU1* lncRNA knockdown. We assessed tumor cell proliferation capacity in vitro using the classic CCK8 assay. As expected, we observed significant inhibition of cell growth at 72–120 hours following the combined treatment of *GAU1* knockdown and H101 infection. After 120 hours of treatment, the cell growth inhibition rates of the combined group reached 62.3–71% (Fig. 3A, B, Inverted triangle) compared to those of the empty vector group of tumor cells (Fig. 3A, B, Square). In comparison, the *GAU1*-silenced group (Fig. 3A, B, Triangle) and the H101-treated group (Fig. 3A, B, cycle) exhibited 18.6% and 31% inhibition, respectively, after 120 hours of treatment. Furthermore, neither combined treatment nor single treatment significantly inhibited NCM460 normal enterocytes growth (Fig. 3C). Next, we tested the ability of tumor cells to form colonies in vitro using a classic colony formation assay after combined treatment with *GAU1* knockdown and H101 infection. Similarly, we observed fewer and smaller cell colonies in the combined group (Fig. 3D, Lane 4) than in the group with *GAU1* silencing alone (Fig. 3D, Lane 2) or the group treated with H101 alone (Fig. 3D, Lane 3). Approximately 77% fewer cell colonies were observed in the combined treatment group than in the empty vector group (Fig. 3E). We also used 5-ethynyl-2’-deoxyuridine (EdU) to label the synthesized DNA with fluorophores and observed cell growth using immunofluorescence technology. We observed 27.2% inhibition after treatment with H101 alone (Fig. 3F, G, Lane 3) and 26.6% inhibition after treatment with *GAU1* alone (Fig. 3F, G, Lane 2) compared to those in the group with empty vector (Fig. 3F, G, Lane 1) in LOVO cells. However, 65% inhibition of LOVO cells was observed in the combined group (Fig. 3F, G, Lane 4). Similarly, we also observed the same trend of inhibition rates in SW620 cells (Fig. 3G, I). These data indicate that the combination of oncolytic adenovirus H101 infection and *GAU1* lncRNA sgRNA significantly inhibited tumor cell growth in vitro.

### The IDTR strategy decreases tumor growth in xenograft models

**Fig. 4:**
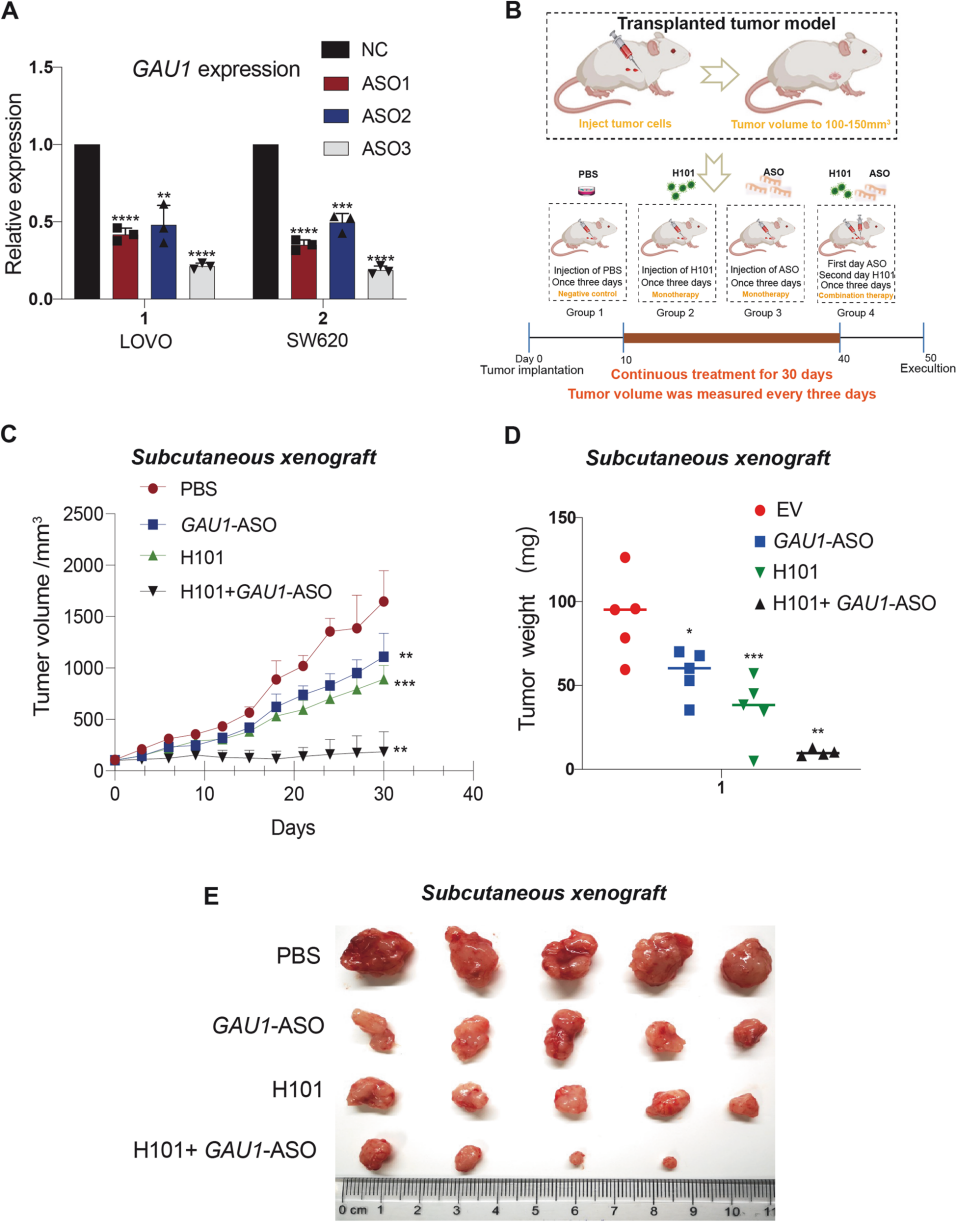
The IDTR strategy decreases tumor growth in xenograft models. **A** Real-time PCR results showed the expression of *GAU1* in SW620 and LOVO cells following *GAU1* ASO transfection. Data were presented as the mean ± SD and the differences between two groups were calculated by unpaired two-tailed *t*-test. N = 3, ****P < 0.0001, ***P < 0.001 and **P < 0.01 compared with negative control (NC). **B** Treatment model of transplanted tumor in mice. When the tumor volume reached 100–150 mm³, the mice were randomly assigned to four groups. The first group was injected with PBS only as a placebo, the second group was injected with H101 alone, the third group was injected with *GAU1* ASO alone, and the fourth group was injected with H101 combined with *GAU1* ASO. All groups were administered according to a 3-day schedule. The single-drug group received intra-tumor injections once every 3 days; the combined group received intra-tumor injection of *GAU1* ASO on day 1 and H101 on day 2. The treatment lasted for a total of 30 days, starting from tumor inoculation, and the mice were euthanized by cervical dislocation after 50 days. **C** Average volume of subcutaneous tumors after treatment with PBS (Cycle), *GAU1*-ASO (Square), H101 (Triangle), or H101 + *GAU1*-ASO (Inverted triangle) was assessed. Values represented the means ± SD for five animals per group and the differences between two groups were calculated by unpaired two-tailed *t*-test. N = 3, ***P < 0.001 and **P < 0.01. **D** Bar graphs showed the weight of allograft tumors in mice that were treated with PBS (Cycle), H101(Square), *GAU1*-ASO (Inverted triangle), and H101 + *GAU1*-ASO (Triangle), respectively (N = 5). Data were presented as the mean ± SD and the differences between two groups were calculated by unpaired two-tailed *t*-test. N = 3, ***P < 0.001, **P < 0.01 and *P < 0.05. **E** Mice xenograft tumors were photographed. After tumor cells were injected into the armpit of mice, they were treated every three days according to the tumor groups and treatment methods. Negative control cells were injected with PBS as described above. After 30 days, the mice were euthanized by cervical dislocation and photographed.

To examine the therapeutic efficacy of the IDTR approach in animal experiments, we chose to use a *GAU1* antisense oligonucleotide (ASO) instead of a *GAU1* sgRNA for increased safety and more efficient silencing. Real-time PCR analysis demonstrated that all three *GAU1* ASOs reduced *GAU1* expression, and *GAU1* ASO-3 exhibited the most effective silencing effect in LOVO and SW620 cells (Fig. 4A). To examine whether the enhanced efficacy in vitro has a similar synergistic effect in vivo, we established a subcutaneous nude mouse xenograft model. When the volume of the tumor reached 100–150 mm^3^, we randomly divided the mice into four groups. The first group received an intratumor injection of PBS only as a placebo, the second group received an intratumor injection of H101 only, the third group received an intratumor injection of *GAU1* ASO only, and the fourth group received an intratumor injection of a combination of H101 and *GAU1* ASO. All groups were subjected to a three-day dosing regimen. The three monotreated groups received one intratumor injection every three days, and the combined group received intratumor injections of the *GAU1* ASO on the first day and H101 on the second day (Fig. 4B). Compared to the PBS control group, monotreatment with either oncolytic adenovirus H101 or *GAU1* ASO moderately inhibited tumor growth, resulting in approximately 33–44% inhibition of tumor growth. However, mice that received the combined treatment of *GAU1* ASO and H101 demonstrated a notably greater reduction in tumor growth (n = 5, *P < 0.05) than those subjected to treatment administered individually, and the overall inhibition rate of tumor growth was approximately 88% (Fig. 4C). After 30 days of continuous treatment, the mice were sacrificed by cervical dislocation, and the tumors were removed and weighed. As expected, subcutaneous xenograft tumors in the combined group were significantly milder than those in the monotreatment groups, the tumor weight was reduced by more than 86% compared to that in the PBS group (Fig. 4D, Triangle), and one mouse tumor completely disappeared following the combination treatment (Fig. 4E). These data demonstrate that the combination of *GAU1* ASO and H101 injection markedly improved the antitumor efficacy of IDTR in vivo.

### There is a lack of cross-talk between *GAU1* knockdown and H101 infection via the IDTR strategy

**Fig. 5:**
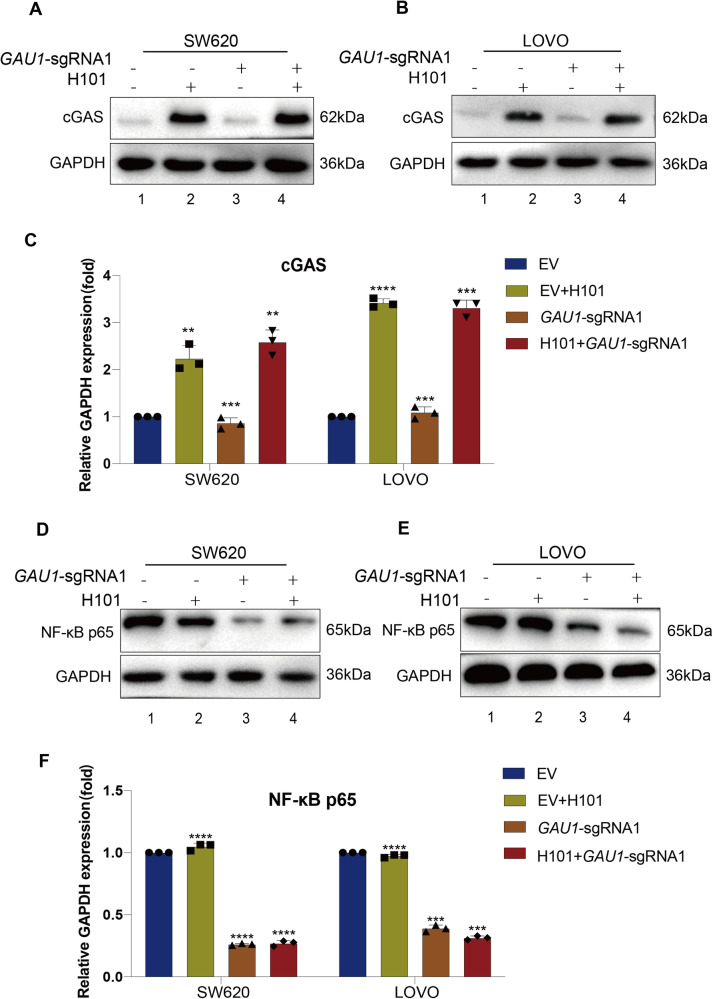
There is a lack of cross-talk between *GAU1* knockdown and H101 infection via the IDTR strategy. **A** Western blot showed the expression of cGAS protein in SW620 cells. The experiments were categorized into empty vector (EV): cells transfected with the empty dCas9-KRAB vector (Lane 1), EV with H101 infection (Multiplicity of Infection (MOI) = 100) after 72 hours (EV + H101) (Lane 2), stable transformation in *GAU1* knockdown (*GAU1* sgRNA) (Lane 3), H101 + *GAU1* sgRNA (Lane 4). **B** Western blot showed the expression of cGAS protein in LOVO cells. **C** Western blot data the expression of cGAS protein was automatically analyzed by ImageJ. GAPDH was used as the internal control for normalization. Data were presented as the mean ± SD and the differences between two groups were calculated by unpaired two-tailed *t*-test. N = 3, ****P < 0.0001, ***P < 0.001 and **P < 0.01. **D** Western blot showed the expression of NF-κB p65 protein in SW620 cells. Treatment methods and control are the same as above. **E** Western blot showed the expression of NF-κB p65 protein in LOVO cells. **F** Western blot data of the expression of NF-κB p65 protein was automatically analyzed by ImageJ. Data were presented as the mean ± SD and the differences between two groups were calculated by unpaired two-tailed *t*-test. N = 3, ****P < 0.0001 and ***P < 0.001. Western blot data was automatically analyzed by ImageJ.

To further investigate whether cross-talk occurred between H101-induced cGAS reactivation and *GAU1* knockdown-induced NF-κB p65 inhibition, we first examined the changes in cGAS expression after *GAU1* knockdown. Western blot analysis showed that *GAU1* knockdown did not change the expression of cGAS (Fig. 5A, Lane 3 and Original full length western blots image 3) compared to H101-induced cGAS activation (Fig. 5A, Lanes 2 and 4 and Original full length western blots image 3) in SW620 cells. Similarly, we observed the same phenomenon in LOVO cells (Fig. 5B and Original full length western blots image 3). The statistical analysis of the western blot data in triplicate further confirmed the above results (Fig. 5C). Next, we detected changes in NF-κB p65 after H101 infection. Western blot assays demonstrated that H101 infection did not change the expression of NF-κB p65 (Fig. 5D, Lane 2 and Original full length western blots image 4) compared to *GAU1* knockdown-induced NF-κB p65 inhibition (Fig. 5D, Lanes 3 and 4 and Original full length western blots image 4) in SW620 cells. Similarly, we observed the same phenomenon in LOVO cells (Fig. 5E and Original full length western blots image 4), and the same trend was observed in the statistical analysis of western blot data in triplicate for the two tumor cell lines (Fig. 5F). These data indicate that there is no cross-talk between *GAU1* knockdown and H101 infection in our IDTR strategy.

### H101 infection increases H3K4me3 levels at the *cGAS* promoter

**Fig. 6:**
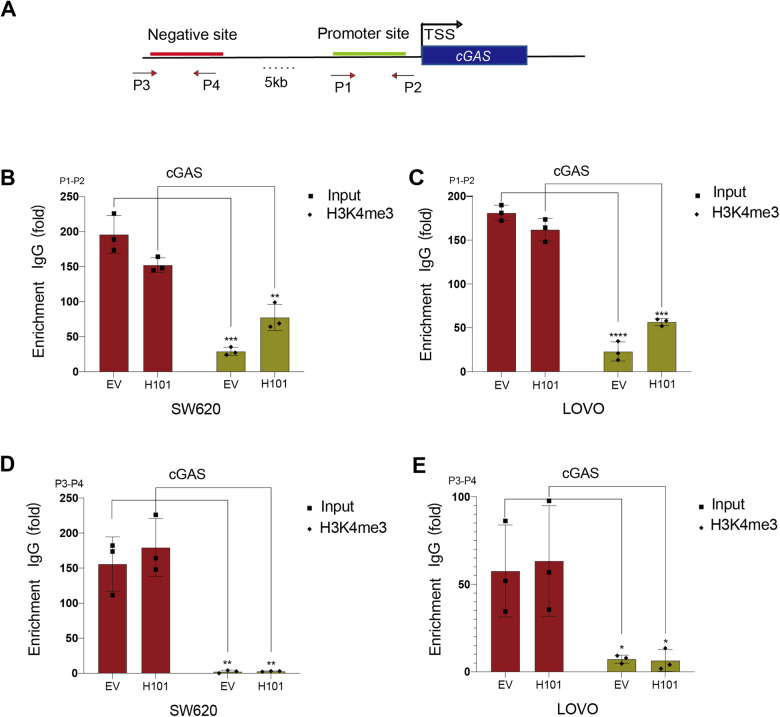
H101 infection increases H3K4me3 levels at the *cGAS* promoter. **A** Schematic of sites in the *cGAS* promoter (P1–P2) and negative site (P3–P4) as detected using the ChIP assay. **B** RT-PCR examination assessed the binding of H3K4me3 to the *cGAS* promoter using samples from the ChIP assay in SW620 cells. P1–P2 represented the *cGAS* promoter site, and P3–P4 indicated the 5000 bp negative control site upstream of the *cGAS* promoter. The value of the IgG group was set as 1. All experiments were performed in triplicate and were presented as the mean ± SEM; N = 3, ***P < 0.001 and **P < 0.01. **C** RT-PCR examination assessed the binding of H3K4me3 to the *cGAS* promoter (P1–P2) using samples from the ChIP assay in LOVO cells. All experiments were performed in triplicate and were presented as the mean ± SEM; N = 3, ****P < 0.0001 and ***P < 0.001. **D** RT-PCR examination assessed the binding of H3K4me3 to the *cGAS* negative site (P3–P4) using samples from the ChIP assay in SW620 cells. N = 3, **P < 0.01. **E** RT-PCR examination assessed the binding of H3K4me3 to the *cGAS* negative site (P3–P4) using samples from the ChIP assay in LOVO cells. N = 3, *P < 0.05.

We next examined the exact mechanism underlying cGAS reactivation after oncolytic adenovirus H101 infection. Detection sites were set up at the *cGAS* promoter (P1–P2) and its upstream negative control site (P3–P4) (Fig. 6A). Chromatin immunoprecipitation (ChIP) assays showed that H101 treatment (MOI of 100) induced significant H3K4 trimethylation (me3) enrichment at the *cGAS* promoter (P1–P2) in SW620 (Fig. 6B) and LOVO tumor cells compared to the nontreated control (Fig. 6C). There was no statistically significant difference in the level of H3K4me3 within the negative control region (P3–P4) (Fig. 6D, E). Taken together, these results suggest that H101 infection activates *cGAS* transcription by increasing the level of H3K4me3 at the *cGAS* promoter in SW620 and LOVO tumor cells.

### *GAU1* knockdown decreases H3K27 acetylation at the *NF-κB p65* promoter

**Fig. 7:**
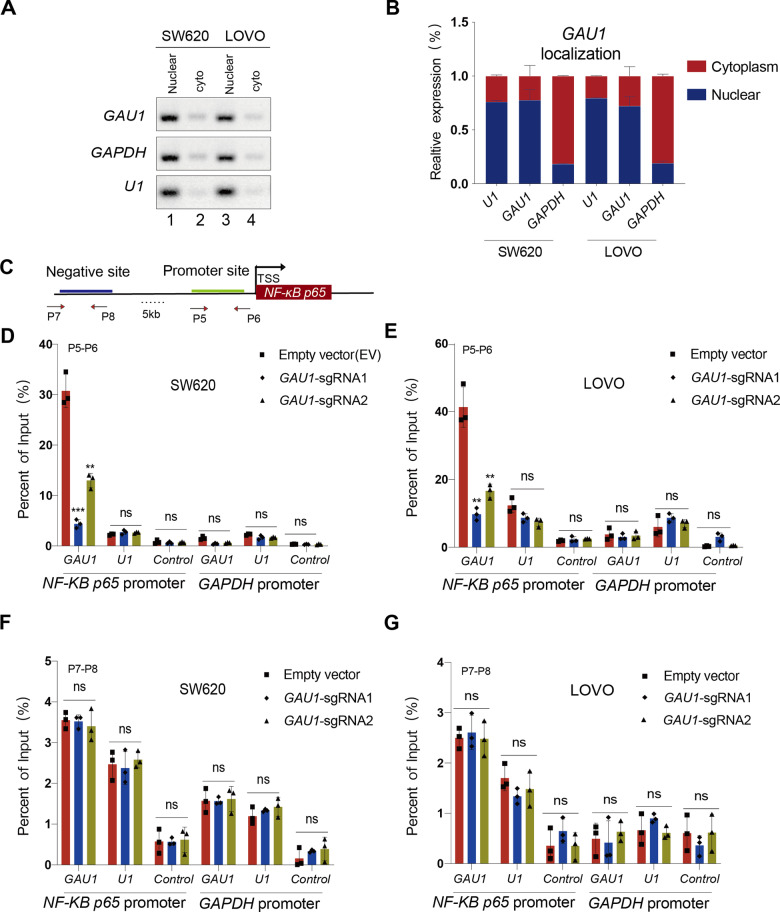
LncRNA *GAU1* binds to the promoter of *NF-κB p65.* **A**, **B** Localization of mature *GAU1* transcripts was examined. *GAU1* was primarily observed in the nucleus. *GAPDH* and *U1* RNA were used as positive controls for the cytoplasmic and nuclear fractions, respectively. **C** Schematic of sites in the *NF-κB p65* promoter (P5–P6) and negative site (P7–P8) as detected using the ChIP and ChIRP assay. **D** RT-PCR examination assessed the binding of *GAU1* to the *NF-κB p65* promoter using samples from the ChIRP assay in SW620 cells. *GAU1* oligo indicated the biotinylated antisense oligonucleotides against the *GAU1* lncRNA. Negative oligo (control) indicated the scrambled oligonucleotides. The *GAPDH* promoter was selected as the negative control. The value obtained for the input was set as 100%. ImageJ was employed for quantifying the binding of *GAU1* to the *NF-κB p65* promoter. P5–P6 represented the *NF-κB p65* promoter site, and P7–P8 indicated the 5000 bp negative control site upstream of *NF-κB p65* promoter. All experiments were performed in triplicate and were presented as the mean ± SEM; N = 3, ***P < 0.001 and **P < 0.01. **E** RT-PCR examination assessed the binding of *GAU1* to the *NF-κB p65* promoter (P5–P6) using samples from the ChIRP assay in LOVO cells. All experiments were performed in triplicate and were presented as the mean ± SEM; N = 3, **P < 0.01. **F** RT-PCR examination assessed the binding of *GAU1* to the *NF-κB p65* negative site (P7–P8) using samples from the ChIRP assay in SW620 cells. **G** RT-PCR examination assessed the binding of *GAU1* to the *NF-κB p65* negative site (P7–P8) using samples from the ChIRP assay in LOVO cells.

We next investigated the precise mechanism underlying NF-κB p65 inhibition after *GAU1* knockdown. We first investigated the location of mature *GAU1* transcripts. By isolating nuclear and cytoplasmic RNA, we found that *GAU1* was predominantly present in the nuclei of LOVO and SW620 cells (Fig. 7A, first panel, Lanes 1 and 3). The statistical analysis of *GAU1* localization in triplicate further confirmed the above results (Fig. 7B). Considering that *GAU1* is primarily localized in the nucleus, it might directly regulate the transcription of *NF-κB p65* by targeting the *NF-κB p65* genomic locus. To investigate whether *GAU1* bound to the *NF-κB p65* promoter, we detected sites within the *NF-κB p65* transcriptional regulatory region, including a promoter site (P5–P6) and a negative control site (P7–P8) (Fig. 7C). Chromatin immunoprecipitation with RNA purification (ChIRP) assays were subsequently performed using biotin-labeled oligomers for chromatin fractionation. The *NF-κB p65* promoter was selected as the detection site, and the *GAPDH* promoter was used as the negative control. ChIRP-qPCR showed that *GAU1* was enriched in the *NF-κB p65* promoter (P5–P6) in SW620 and LOVO cells (Fig. 7D, E, Square). However, *GAU1* enrichment at the *NF-κB p65* promoter significantly decreased after *GAU1* knockdown (Fig. 7D,E, Triangle and Diamond). No notable difference was detected in the negative control site (P7–P8) following *GAU1* knockdown in SW620 (Fig. 7F) and LOVO cells (Fig. 7G). These results show that *GAU1* is a nuclear lncRNA that can directly bind to the *NF-κB p65* promoter in SW620 and LOVO cells.

**Fig. 8:**
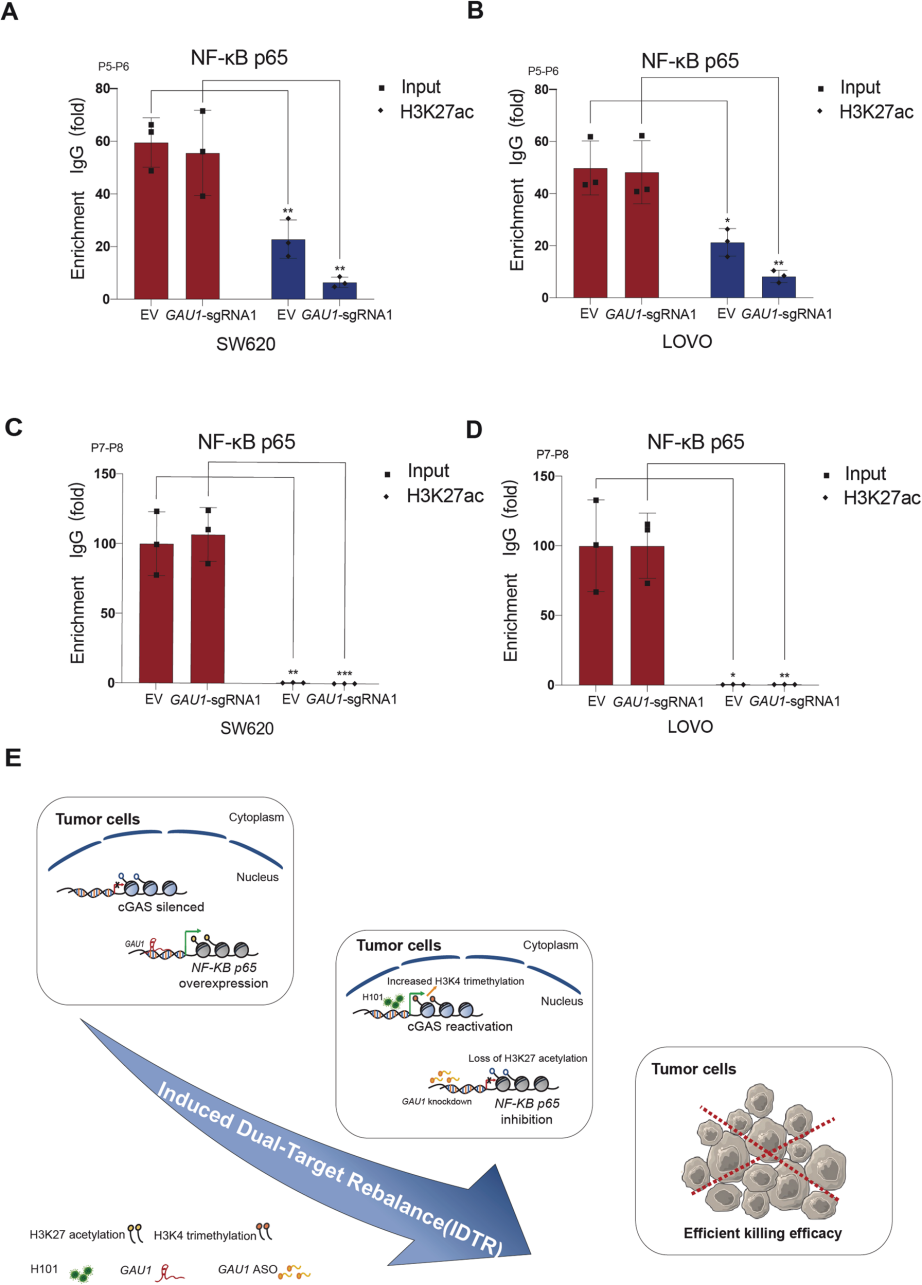
*GAU1* knockdown decreases H3K27 acetylation at the *NF-κB p65* promoter. **A** Real time-PCR examination assessed the binding of H3K27ac to the *NF-κB p65* promoter using samples from the ChIP assay in SW620 cells. P5–P6 represented the *NF-κB p65* promoter site, and P7–P8 indicated the 5000 bp negative control site upstream of *NF-κB p65* promoter. The value of the IgG group was set as 1. All the experiments were performed in triplicate and were presented as the mean ± SEM. N = 3, ***P < 0.001, **P < 0.01 and *P < 0.05. **B** Real time-PCR examination assessed the binding of H3K27ac to the *NF-κB p65* promoter (P5–P6) using samples from the ChIP assay in LOVO cells. The value of the IgG group was set as 1. All the experiments were performed in triplicate and were presented as the mean ± SEM. N = 3, **P < 0.01 and *P < 0.05. **C** Real time-PCR examination assessed the binding of H3K27ac to the negative site (P7–P8) using samples from the ChIP assay in SW620 cells. The value of the IgG group was set as 1. All the experiments were performed in triplicate and were presented as the mean ± SEM. N = 3, ***P < 0.001 and **P < 0.01. **D** Real time-PCR examination assessed the binding of H3K27ac to the negative site (P7–P8) using samples from the ChIP assay in LOVO cells. The value of the IgG group was set as 1. All the experiments were performed in triplicate and were presented as the mean ± SEM. N = 3, **P < 0.01and *P < 0.05. **E** Induced Dual-Target Rebalance (IDTR) strategy diagram was illustrated. Oncolytic adenovirus H101 reactivated *cGAS* transcription by increasing H3K4me3 at the *cGAS* promoter and *GAU1* ASO inhibited *NF-κB p65* transcription by decreasing H3K27ac at the *NF-κB p65* promoter, promoting antitumor efficacy.

We further elucidated how *GAU1* could modulate *NF-κB p65* transcription. We detected histone modifications at the *NF-κB p65* promoter site (P5–P6) and negative control site (P7–P8) by ChIP‒qPCR (Fig. 7C). ChIP‒qPCR showed that *GAU1* knockdown significantly decreased the level of H3K27 acetylation (ac) at the *NF-κB p65* promoter (P5–P6) in SW620 (Fig. 8A) and LOVO (Fig. 8B) cells. We did not observe significant differences in H3K27ac in the negative control (P7–P8) (Fig. 8C, D). These data suggest that *GAU1* knockdown diminishes the level of H3K27ac at the *NF-κB p65* promoter.

## Discussion

In contrast to conventional single-target therapies, simultaneous targeting of various tumor-specific abnormalities has become an important and attractive approach for accessing antitumor therapies [21]; however, discovering suitable targets and effective methods is a global challenge for targeted tumor therapy. In this study, for the first time, we established a novel IDTR strategy for the simultaneous reactivation of silenced cGAS using the oncolytic adenovirus H101 and the inhibition of NF-κB p65 overexpression with *GAU1* ASO, ultimately resulting in efficient antitumor therapeutic efficacy in vitro and in vivo (Fig. 8E). We also revealed for the first time an alternative therapeutic mechanism in which oncolytic adenovirus H101 could reactivate *cGAS* transcription by increasing H3K4me3 at the *cGAS* promoter and *GAU1* knockdown could inhibit *NF-κB p65* transcription by decreasing H3K27ac at the *NF-κB p65* promoter, thereby providing a suitable dual-target and effective IDTR approach for promoting antitumor efficacy.

Notably, NF-κB p65 and cGAS are two frequently dysregulated cellular regulatory factors in tumors. Overexpression of NF-κB p65 and silenced expression of cGAS are often critical triggers contributing to tumor initiation, drug resistance, and treatment evasion [22–24]. Inhibition of NF-κB p65 overexpression could restrain tumor cell proliferation and invasion [14]. On the other hand, reactivation of cGAS expression enhances immune activity, facilitating the recognition and clearance of tumor cells and thereby increasing overall treatment efficacy [25, 26]. However, to our knowledge, reactivation of silenced cGAS expression and inhibition of NF-κB p65 overexpression have not been carried out simultaneously in tumor therapy. In this study, we chose colorectal cancer patients exhibiting simultaneous NF-κB overactivation and cGAS inactivation as the research model. We employed two classic colorectal cancer cell lines, SW620 and LOVO, and conducted both in vitro and in vivo experiments. Therefore, we successfully established a novel IDTR antitumor approach that restored NF-κB p65 and cGAS abnormalities at the same time and showed efficient therapeutic efficacy in tumors. Since each type of tumor has varying overexpressed and low-expressed targets, it is interesting to explore different combinations of over- and low-target effects by utilizing our IDTR strategy to obtain better antitumor therapeutic efficacy.

Notably, both cGAS and NF-κB p65 are key factors involved in immune regulation [27]. Activation of cGAS induces the production of type I interferons (IFNs), which initiate innate immune responses against tumor cells [28]. cGAS activation promotes the activation and recruitment of immune cells such as natural killer (NK) cells, dendritic cells (DCs), and T cells, enhancing antitumor immune responses [29, 30]. On the other hand, NF-κB p65 overactivation upregulates the expression of genes involved in cell survival, proliferation, and antiapoptotic pathways, promoting tumor cell survival and proliferation [31, 32]. Abnormal activation of NF-κB p65 also induces the production of proinflammatory cytokines and chemokines, creating a protumorigenic inflammatory microenvironment that supports tumor growth and progression [33]. Moreover, NF-κB p65 overactivation can induce the expression of immune checkpoint molecules such as PD-L1 on tumor cells or immune cells, contributing to immune evasion by suppressing antitumor immune responses [34, 35]. In this study, we found that simultaneously activating aberrantly inactivated cGAS and inhibiting the overactivation of NF-κB p65 in tumors produces a potent synergistic antitumor effect. A compelling hypothesis is that simultaneously restoring the function of these two key factors may involve the coordinated regulation of tumor immune cascade pathways, providing a more comprehensive intervention in tumor cell immune evasion mechanisms and thereby enhancing the antitumor effect. An intriguing research direction going forward is to focus on elucidating the detailed molecular mechanisms underlying the synergistic antitumor effects achieved by correcting abnormalities in both cGAS and NF-κB p65.

Studies have shown that poly(rC)-binding protein 1 (PCBP1) is recruited to the cGAS protein to increase its activity and enhance the affinity of cGAS for its ligand during viral infection [36]. Recent investigations have proposed that viral control over the host might be linked to viral transcriptional regulators (vTRs) [37]. Intriguingly, the E1A protein encoded by the adenovirus genome could interact with retinoblastoma protein (pRB) and EP300 to activate the transcription of multiple genes that promote the cell cycle and proliferation [38]. Our previous study showed that an oncolytic adenovirus called H101 has been clinically approved for the treatment of malignancies because it selectively kills tumor cells through viral oncolysis and does not have significant cytopathic effects on normal cells [21]. H101 infects tumor cells by releasing its genome, which induces tumor cell lysis and apoptosis [39]. Therefore, these studies led to the hypothesis that the oncolytic adenovirus H101 is likely to reactivate cGAS. Based on this hypothesis and subsequent validation, for the first time, we discovered that oncolytic adenovirus H101 could actually induce the reactivation of cGAS. We first revealed that cGAS reactivation was caused by increased H3K4 trimethylation of the *cGAS* promoter. We speculate that the dynamics of histone modification may be associated with vTRs derived from H101. However, we have yet to determine which vTRs are involved in the transcriptional regulation of *cGAS*, which will be an interesting avenue for our future research. It should also be emphasized that we cannot theoretically eliminate other chromatin modifications involved in cGAS reactivation in a positive or negative manner. Thus, it would be of great interest to focus on the identification of other causes to better understand the reactivation of cGAS.

NF-κB p65 proteins are mainly localized in the cytoplasm and form an inactive trimeric complex with the inhibitory protein IκB [40]. Activation of the NF-κB p65 protein occurs mainly through phosphorylation of the IκB protein, which is ubiquitinated and degraded to release the active NF-κB p65 protein [41, 42]. *NKILA* lncRNA can bind to NF-κB/IκB and shield the phosphorylation site of I-κB, thereby inhibiting IκB kinase (IKK)-induced I-κB phosphorylation, which in turn inhibits the activation of NF-κB proteins and suppresses cancer metastasis [43]. Many studies have focused on the regulation of NF-κB p65 protein activity by lncRNAs in the cytoplasm through influencing IκB dissociation. However, in this study, for the first time, we showed that the knockdown of the lncRNA *GAU1* by utilizing ASOs or single-guide RNAs (sgRNAs) could inhibit the overexpression of NF-κB p65 in human colorectal tumors. *GAU1*, which was initially discovered and named in our prior research, was first identified in neuroblastoma and retinoblastoma [44]. This prompted us to delve deeper into the underlying mechanisms by which *GAU1* promotes the transcriptional activation of *NF-κB p65*. Upon further investigation of the underlying mechanisms, we first revealed that NF-κB p65 inhibition was caused by decreased H3K27 acetylation of the *NF-κB p65* promoter. Notably, the ability of *GAU1* to inhibit NF-κB p65 overexpression is unlikely to be completely regulated by *GAU1* due to the multiple functions of lncRNAs. Thus, further exploration of the precise role of *GAU1* in human colorectal tumorigenesis is of potential interest.

Notably, in this study, the combination of H101 and *GAU1* silencing was chosen as the treatment strategy for tumors. Although we observed a certain degree of tumor suppression with H101 infection, its therapeutic efficacy remains suboptimal [45]. Therefore, enhancing the therapeutic efficacy of H101 is also an important issue that needs to be considered. Concurrent administration of si*GNAQ* and H101 significantly impedes the proliferation of UM cells and triggers apoptosis [46]. Additionally, intratumoral injection of H101 in conjunction with concurrent chemoradiotherapy substantially enhances the therapeutic efficacy against advanced cervical cancer, leading to improved patient survival rates [47]. These studies indicate that combination therapy is an effective approach for enhancing the therapeutic efficacy of H101. In this study, we found that when H101 was combined with *GAU1* silencing, the therapeutic efficacy against tumors was significantly enhanced. Compared to the monotherapy group, the combination therapy group showed a 2–3.5-fold increase in the inhibition of tumor growth. This combined approach also provides innovative insights for our subsequent development of targeted antitumor treatment strategies.

Notably, CRISPR/dCas9 is internationally recognized as a powerful gene knockdown tool that enables the establishment of stable knockdown states in cells [48]. We confirmed the effective knockdown of *GAU1* in vitro, ensuring reliable experimental outcomes. However, we chose to use *GAU1*-ASO for the treatment of mouse xenograft tumors because it represents a safer and convenient alternative method. ASO drugs exclusively involve posttranscriptional interference, disrupting RNA levels without altering the genome, and are also recognized as clinically accepted gene silencing therapy approaches [49, 50]. This provides potential for the development of clinical-grade *GAU1*-ASO drugs. Intratumoral injection allows for the drug to directly target the tumor site, improving drug stability and enabling maximum therapeutic efficacy with smaller doses [51]. In contrast, intravenous administration of drugs requires circulation throughout the body to reach the tumor site, which can lead to drug degradation and accumulation at other sites. However, to ensure the effectiveness of the treatment, it is necessary to increase the dosage of the drug, potentially increasing the risk of toxicity due to higher drug concentrations.

## Materials and methods

### Cell culture

The human cell lines SW620 (ATCC, CCL-227), LOVO (ATCC, CCL-229), NCM460 (Shanghai Cell Bank, TCH-C450), and 293 T (ATCC, CRL-3216) were cultured in DMEM (Gibco, Waltham, MA) supplemented with 10% certified heat-inactivated fetal bovine serum (Yeasen, Shanghai, China), 100 U/ml penicillin, and 100 mg/ml streptomycin at 37 °C in a humidified atmosphere containing 5% CO_2_. The recombinant adenovirus H101 was generously provided by Shanghai Sunway Biotech (Shanghai, China) and was stored at −80 °C as recommended.

### Reverse transcription-quantitative polymerase chain reaction (RT-qPCR) assay

Total RNA was extracted from LOVO and SW620 cells using TRI-REAGENT (Invitrogen, Waltham, MA). The RNA was then reverse transcribed using qPCR SYBR Green Master Mix (Yeasen, Shanghai, China) and analyzed with a LightCycle96 quantitative PCR system (Roche, Basel, CH). The relative mRNA expression levels of genes were calculated using the 2^-ΔΔCt^ method after normalization to the expression of *GAPDH*, which served as an internal loading control. To detect the expression levels of *GAU1, cGAS*, and *NF-κB p65*, we designed qPCR primers, which were synthesized by Sangon (Shanghai, China), according to the manufacturer’s protocol and a previous study. The primer sequences can be found in the supplementary materials (Table S1).

### Western blot analysis

Cells were collected at the specified times and washed twice with PBS. The cell extracts were prepared using lysis buffer and then centrifuged at 13,000 × *g* for 30 minutes at 4 °C. The protein concentration was quantified using a BCA kit (Yeasen, Shanghai, China). The supernatant was collected, and 5× SDS protein loading buffer was added in proportion, followed by incubation at 100 °C for 10 minutes. Proteins were separated on a 10–12% SDS‒PAGE gel and then electrophoretically transferred to polyvinylidene difluoride (PVDF) membranes (Millipore, Boston, MA). The membranes were blocked with 5% nonfat milk in Tris‐buffered saline supplemented with 0.1% Tween 20 at room temperature for 2 hours. The primary antibody was diluted with 1× TBST according to the instructions and incubated overnight at 4 °C. Subsequently, the membranes were incubated with secondary antibodies for another 2 hours at room temperature. Immunoblots were visualized using an enhanced chemiluminescence detection kit (Yeasen, Shanghai, China) with a chemiluminescence imaging analysis system (Tanon, Shanghai, China). Relative integrated density values were calculated using ImageJ software. The following antibodies were used: anti-cGAS, cGAS (E5V3W); rabbit mAb #79978 (CST, Boston, MA); anti-NF-κB, p65 (D14E12); XP® rabbit mAb #8242 (CST, Boston, MA); and GAPDH (Sigma‒Aldrich, Louis, MI).

### Chromatin immunoprecipitation (ChIP) assay

ChIP was performed using an EZ-Magna ChIP A/G kit (Millipore, Boston, MA) following the manufacturer’s instructions and a previously reported protocol. The anti-H3K27ac and H3K4me3 antibodies used for RNA-ChIP were also used for ChIP. Normal rabbit IgG was used as a negative control. Anti-H3K27ac: Acetyl-Histone H3 (Lys27) (D5E4) XP® Rabbit mAb #8173(CST, Boston, MA); Anti-H3K4me Tri-Methyl-Histone H3 (Lys4) (C42D8) Rabbit mAb #9751(CST, Boston, MA); Anti- IgG: Rabbit (DA1E) mAb IgG XP® Isotype Control #3900(CST, Boston, MA). The primer sequences are listed in the supplementary materials (Table S1).

### Nuclear fractionation

Cells were collected at the specified times and washed twice with PBS. Subsequently, 10^7^ cells were harvested and resuspended in 1 ml of ice-cold DEPC-PBS, 1 ml of buffer C1 (1.28 M sucrose, 40 mM Tris–HCl [pH 7.5], 20 mM MgCl_2_, 4% Triton X-100), and 3 ml of RNase-free water. The plates were then incubated for 15 minutes on ice. Afterward, the cells were centrifuged for 15 minutes at 3000 rpm. The resulting supernatant containing the cytoplasmic component and the pellet containing the nuclear fraction were both retained for RNA extraction.

### CRISPR/dCas9-KRAB-mediated gene editing and cell transfection

Single guide RNA sequences were designed based on the promoter of the human *GAU1* lncRNA and predicted using the online tool created by Prof. Zhang (http://crispr.mit.edu/). The sgRNA sequences can be found in the supplementary materials, specifically in the primer table. The targeting plasmids and packaging plasmids were cotransfected into 293 T cells using Lipofectamine 3000 (Invitrogen, Waltham, MA). After 48 or 72 hours, the supernatant was purified, ultracentrifuged, and collected for cell infection. Prior to transfection, 1 × 0^5^ LOVO and SW620 cells were cultured in 6-well plates with 2 ml of complete medium for 24 hours until they reached 50% confluence. The vectors carrying sgRNA were transfected into the LOVO and SW620 cell lines using Lipofectamine 3000 (Invitrogen, Waltham, MA) and cultured in serum-free Opti-MEM according to the manufacturer’s instructions. Puromycin (5 µg/ml) was added 72 hours after infection, and the surviving cells were collected for selection.

### *GAU1* antisense oligonucleotides

Cells were transfected at 60–80% confluence with 20–50 nmol/L antisense oligonucleotides (ASOs) (RiboBio, Guangzhou, China) targeting *GAU1* using Lipofectamine 3000 (Invitrogen, Waltham, MA) according to the manufacturer’s instructions. As a control, cells were transfected with scrambled ASO. Forty-eight hours after transfection, the cells were harvested for further analysis. The ASO sequences are listed in the supplementary materials (Table S1).

### ChIRP assay

Biotin-labeled probes against *GAU1* were designed according to an online tool (www.singlemoleculefish.com). Probes against LacZ RNA were used as negative controls. Probes against U1 were used as positive controls. A total of 2 × 10^7^ SW620 and LOVO cells were resuspended in precooled PBS buffer and crosslinked with 3% formaldehyde, and the reaction was quenched with glycine. The cells were then pelleted at 1000 × *g* for 10 min and resuspended in swelling buffer containing 0.1 M Tris (pH 7.0), 10 mM KOAc, and 15 mM MgOAc. 1% NP-40, 1 mM DTT, 1 mM PMSF, complete protease inhibitor, and 0.1 U/µl Superase-in for 10 min on ice. The nuclei were further resolved in 50 mM Tris 7.0, 10 mM EDTA, and 1% SDS, and DTT, PMSF and Superase-in were added. The chromatin was then sonicated to 100–500 bp in size and diluted in 500 mM NaCl, 1% SDS, 100 mM Tris 7.0, 10 mM EDTA, and 15% formamide. Prebinding probe oligos were added to streptavidin beads. The beads were mixed with the cell lysate and hybridized at 37 °C overnight on an end-to-end shaker. Subsequently, the beads were washed five times with 1 ml of prewarmed wash buffer for 5 min per wash. Then, 100 µl of elution buffer containing 50 mM NaHCO_3_, 1% SDS, 200 mM NaCl, 20 U of RNase A and 20 U of RNase H was added, and the mixture was eluted at 37 °C for 1 h. Subsequently, the supernatant was collected, 10 µl of protease K (Thermo Fisher, Waltham, MA) was added, and the mixture was incubated at 55 °C for 2 h. DNA was then extracted with phenol:chloroform:isoamyl and precipitated with ethanol at −80 °C. Furthermore, the purified DNA was subjected to qPCR analysis. The primer sequences are listed in the supplementary materials (Table S1).

### Cotreatment of tumor cells with *GAU1* sgRNAs/ASOs and oncolytic adenovirus H101

#### Method 1

LOVO and SW620 cell lines stably transfected with *GAU1* CRISPR/dCas9-KRAB and empty vector were constructed, 30–50% of the above cells were transferred to a 6-well plate, and the cells were infected with H101 at a MOI of 100. The control group included cells transfected with empty vector or PBS.

#### Method 2

LOVO and SW620 cells were combined in a 6-well plate with 30–50% Lipofectamine 3000 (Invitrogen, Waltham, MA) and transfected with 50 nmol/negative control ASO or *GAU1* ASO according to the manufacturer’s instructions (Invitrogen, Waltham, MA). After incubation overnight, the cells were infected with H101 at an infection factor of 100 MOI. The control group included cells transfected with negative control ASO or PBS.

### Cell counting kit-8 (CCK-8) assay

For the CCK-8 assay, the transfected cells (1 × 10^3^ cells/well) were seeded into 96-well plates in quintuplicate and cultured until they were entirely adherent. CCK-8 solution (10 μl, Yeasen, Shanghai, China) was added to each well at 0, 24, 48, 72, and 96 hours. After 2 hours of incubation, the absorbance at 450 nm was measured on a microplate reader (SpectraMax iD3).

### Colony formation assay

The cells were also counted and adjusted to 1000 cells per well in 6-well plates and then placed into a humidified incubator for 1 week. Then, the cells were washed twice in phosphate-buffered saline (PBS), fixed with 70% methanol for 30 min and stained for 30 min with crystal violet. The colonies were counted in assays. Finally, GraphPad Prism 8.0 was used to analyze the number of cell colonies in each well.

### EdU assay

For EdU labeling, 1 × 10^5^ cells were seeded in each well of a 6-well plate in Dulbecco’s modified Eagle’s medium (DMEM) supplemented with 10% fetal bovine serum (FBS). Twenty-four hours later, 10 μM EdU (Beyotime, Shanghai, China) was added to the medium. After another 72 hours, the cells were fixed for EdU staining or for further tests. For EdU staining, the cells were fixed with methanol, washed twice with phosphate-buffered saline (PBS), incubated in 3% bovine serum albumin (BSA) in PBS, and then incubated in 0.5% Triton® X-100 in PBS for 20 min at room temperature. The cells were then incubated with freshly made Click-iT reaction cocktail, which contained azide-conjugated Alexa 594 (Invitrogen, Waltham, MA), for 30 min at room temperature in the dark. The cells were further stained with 4′,6-diamidino-2-phenylindole (DAPI, for nuclear staining, 1 μg/ml, Sigma‒Aldrich, Louis, MI) and then mounted in standard mounting media. The stained cells were examined with a Nikon camera. To determine the percentage of EdU-positive cells, the number of red fluorescent (Alexa 594-stained) cells was divided by the number of blue fluorescent (DAPI-stained) cells. The experiment was performed in triplicate, and the data are presented as the average of three independent experiments.

### Tumor xenograft model in nude mice

A xenograft model of LOVO cell tumors was established by injecting 5 × 10^6^ cells into the right armpit of female nude mice aged 4-6 weeks. When the tumors reached the desired mean tumor volume (100–125 mm^3^) (length (mm) × width (mm) ^2^/2), the animals were randomly divided into four groups. The *GAU1* ASO + H101 group was injected with 20 µg *GAU1* ASO via the intertumoral route on Days 1, 4, 7, 10, 13, 16, 19, 22, 25, and 28, and adenovirus H101 was injected via the intertumoral route with 1 × 10^8^ PFU/mouse on Days 2, 5, 8, 11, 14, 17, 20, 23, 26, and 29. The *GAU1* ASO group was injected with 10 µg of *GAU1* ASO 10 times. The H101 adenoviral group was subjected to 10 intertumoral injections of H101, and the control group was injected with PBS 10 times. Tumor size was measured every 3 days with a Vernier caliper. The animal experiments were carried out in accordance with the guidelines of the SPF Animal Center of Tongji University.

### Statistical analysis

All experiments were performed in triplicate, and the data are expressed as the mean ± SD. The comparative CT method was applied in the quantitative real-time RT‒PCR assay according to the delta‒delta CT method. The data are presented as the mean ± SD, the differences between two groups were calculated by unpaired two-tailed t tests, and the results were considered to be statistically significant. If *P* < 0.05, it is considered to have statistical difference, it is represented by ‘*’; If *P* < 0.01, it is indicated by ‘**’; If *P* < 0.001, it is indicated by ‘***’; If *P* < 0.0001, it is indicated by ‘****’.

### Supplementary information

Supplementary information

Original full-length western blots

## Data Availability

The data that support the findings of this study are available on request from the corresponding author, He Zhang, upon reasonable request. The original western blot images are provided in the Supplementary File.
