# Supplementary material for: Induced dual-target rebalance simultaneously enhances efficient therapeutical efficacy in tumors
Source: Cell Death Discov. 2024 May 23;10:249. doi: 10.1038/s41420-024-02018-y (PMC11116470; doi:10.1038/s41420-024-02018-y)
Supplement: Supplementary file 1 — Supplementary information [file 41420_2024_2018_MOESM1_ESM.pdf]

Supplementary information

Supplemental Table 1: Primers, oligos, sgRNAs used in the experiment.

	**PCR**
GAPDH	TCCAAAATCAAGTGGGGCGA
	TGATGACCCTTTTGGCTCCC
GAPDH - ChIRP	TCGGAGTCAACGGGTGAGTT
	CTACCCTGCCCCCATACGA
Negative probes-ChIP	GGAGCCATTGTATGTCGTGC
	ACCCCAAAGTCCTACCCAGT
GAU1 primer	TATCACCGGAAGCACGCATT
	TTTGTGCTTTGGGAAGGGCT
GAU1 sgRNA1	ACATCTGAATAAACGCTACG
GAU1 sgRNA2	TGTGGAGACCACCCGTAAAC
cGAS primer	GGAAGCAACTACGACTAAAGC
	CGATGTGAGAGAAGGATAGCC
cGAS promoter ChIP	CTCTCGGATGCTGAGGTTAC
	CGAAGTTTCTAACTCTCCAGGC
cGAS negative ChIP	CCAGATTGCTGGGATTACAGG
	CAACCCAGCAAGACCTGGTC
NF-κB primer	GGCGAATGGCTTTACTTTAGC
	CAGAAAGGGGCGGAAGAC
NF-κB promoter ChIP	TCTAAACCTTAGCAGACAGCAC
	GCAGACACCTGTACCTCTAA
NF-κB negative ChIP	AATCAGCCAGATGACATAAGC
	CACCACTGGAGCTATATTCTAA
ChIRP-probes	ATCTATGAAGCTGGGTGGAG
	GTGAGAGAGGGAGATTCCAT
	GTTGTTGACTGAAATGAAGG
	GCGCTATGAAGAAACTTGGC
	AGAGATACAAGCCACATGGG
Control-probe	CAGTGAATCCGTAATCATG
