# Supplementary material for: Induced dual-target rebalance simultaneously enhances efficient therapeutical efficacy in tumors
Source: Cell Death Discov. 2024 May 23;10:249. doi: 10.1038/s41420-024-02018-y (PMC11116470; doi:10.1038/s41420-024-02018-y)

Figure1 western blot

Original western blots image 1

NCM460 LOVO SW620

cGAS

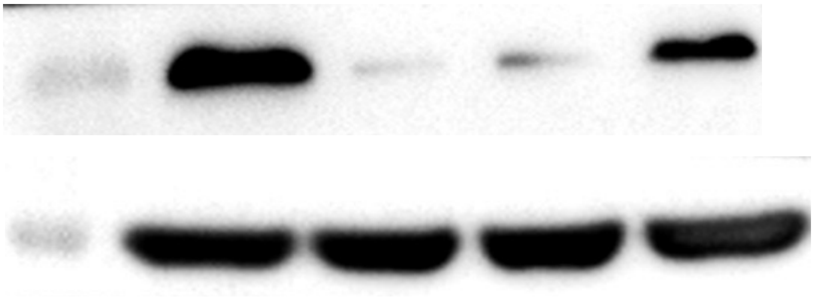

62kDa

GAPDH

36kDa

1 2 3

NCM460 LOVO SW620

NF-κB p65

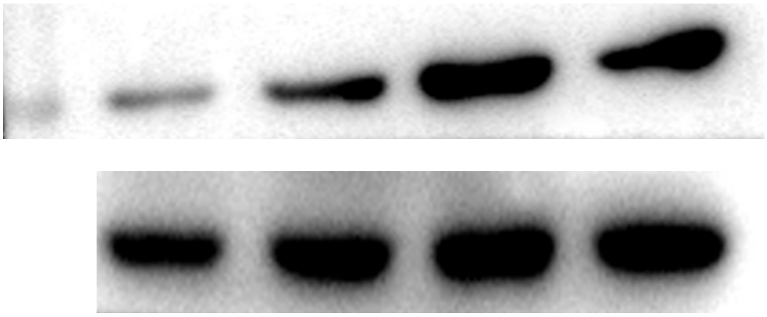

65kDa

GAPDH

36kDa

1 2 3

Figure2 western blot

Original western blots image 2

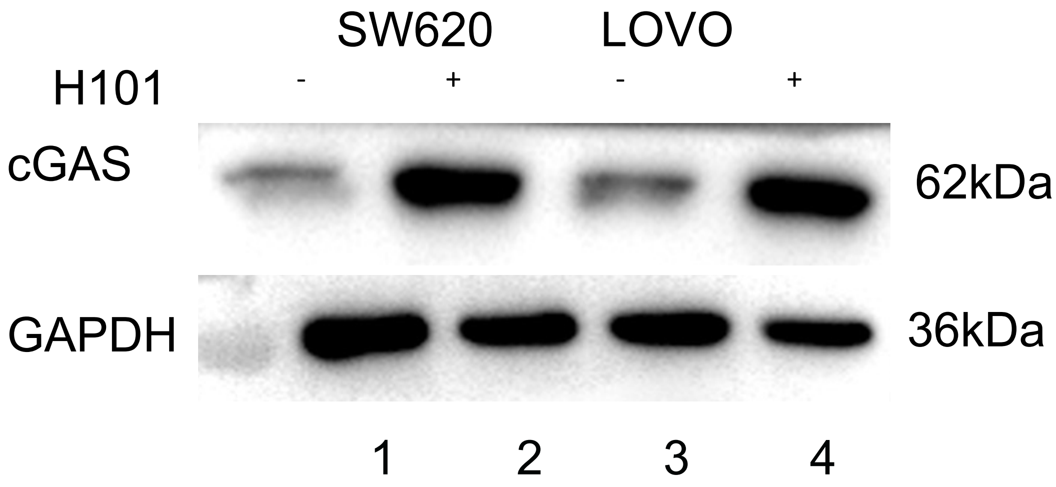

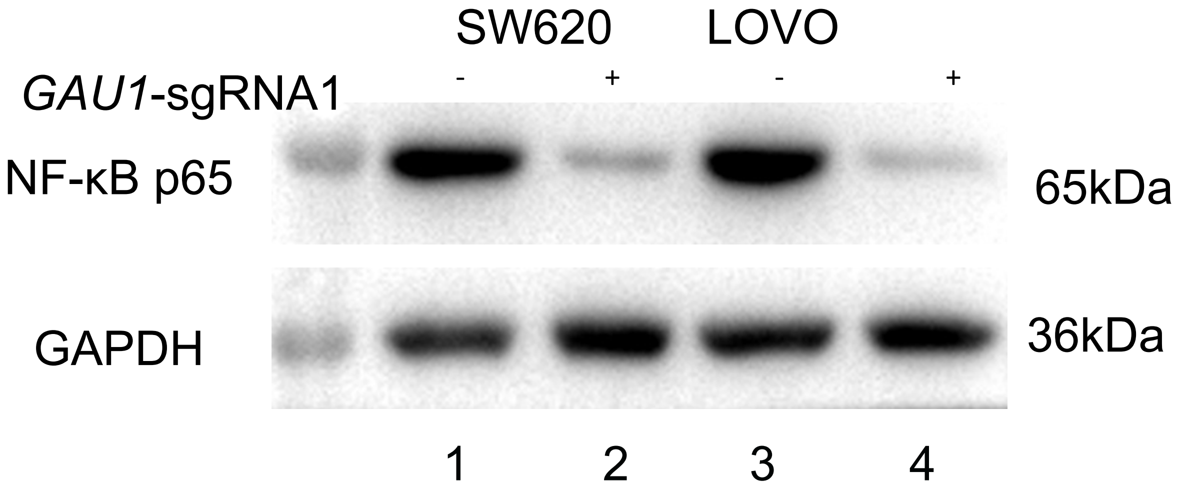

Figure5 western blot

Original western blots image 3

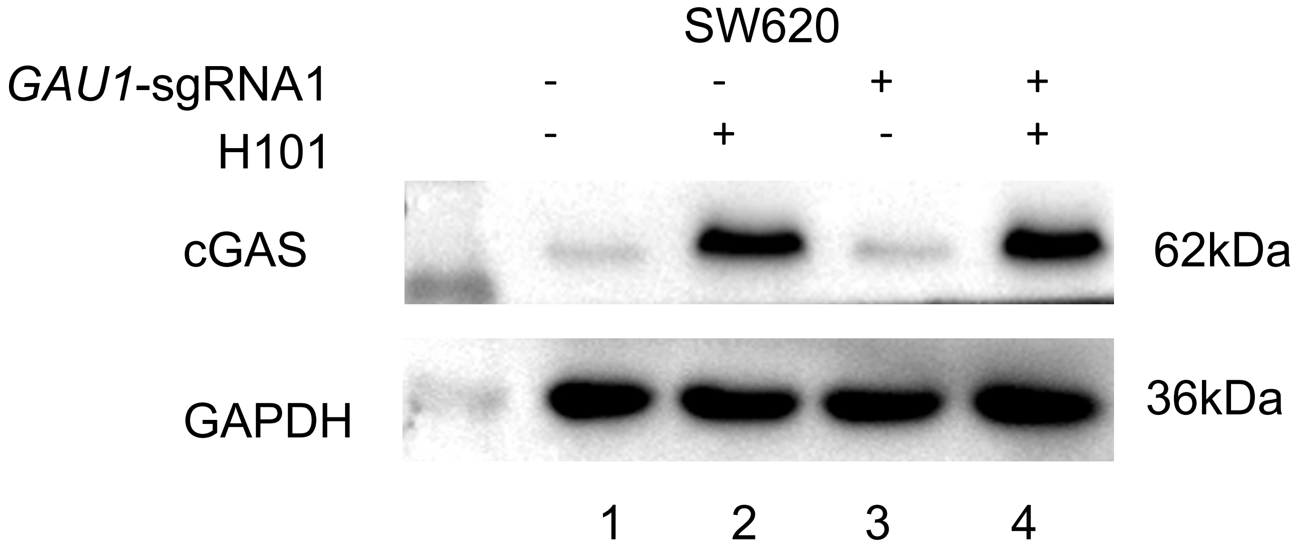

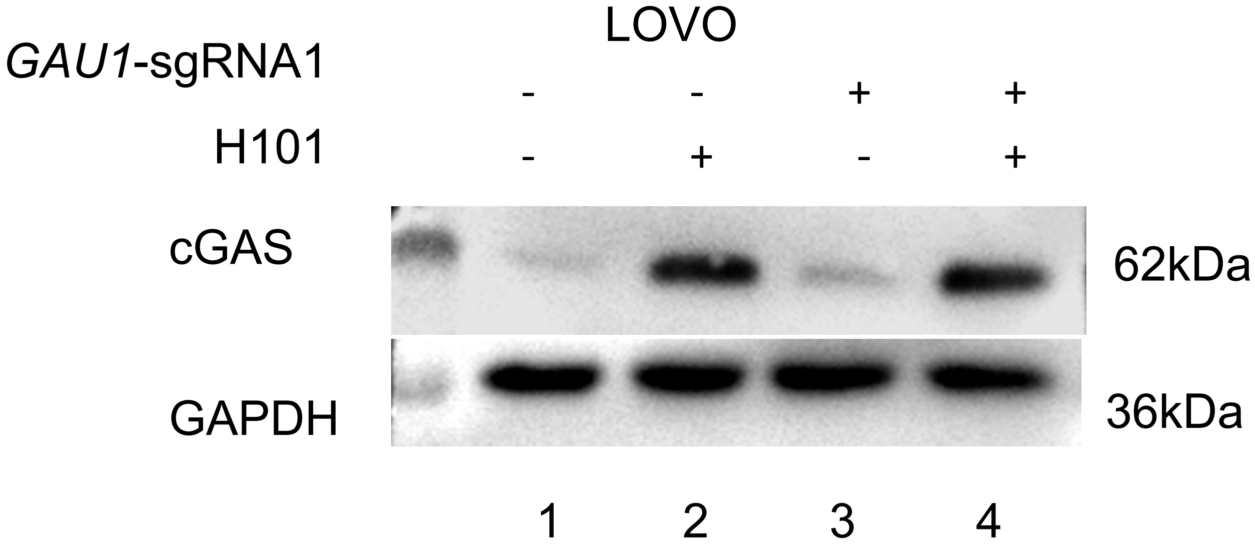

Figure5 western blot

Original western blots image 4

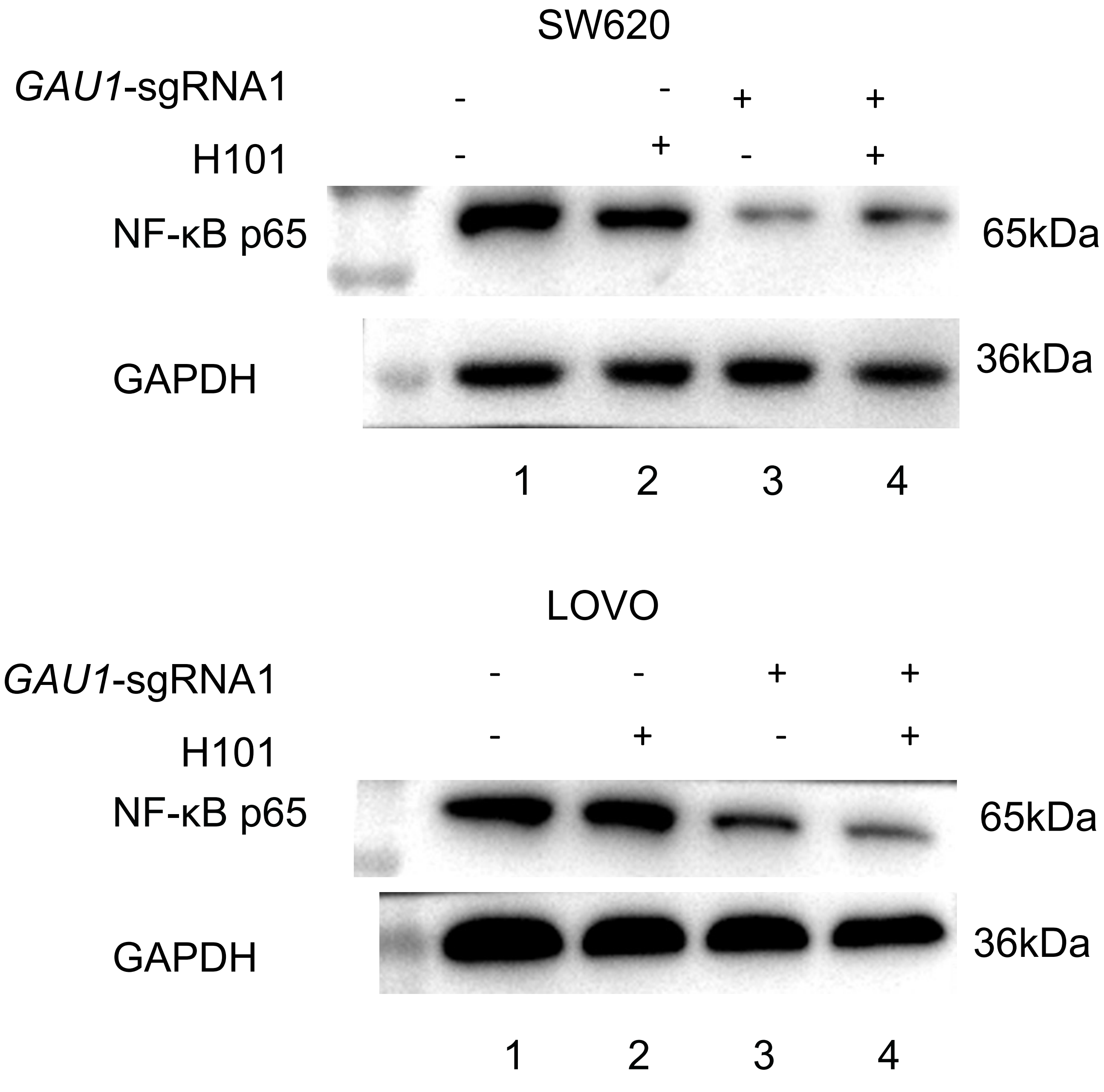

Supplement: Supplementary file 2 — Original full-length western blots [file 41420_2024_2018_MOESM2_ESM.pdf]
